# Feasibility of an integrated multidisciplinary geriatric rehabilitation programme for older stroke patients: a process evaluation

**DOI:** 10.1186/s12883-020-01791-4

**Published:** 2020-05-29

**Authors:** Tom P. M. M. Vluggen, Jolanda C. M. van Haastregt, Jeanine A. Verbunt, Caroline M. van Heugten, Jos M. G. A. Schols

**Affiliations:** 1 Department of Health Services Research, Maastricht University, Maastricht, The Netherlands; 2 Care and Public Health Research Institute, Maastricht University, Maastricht, The Netherlands; 3 Adelante, Centre of Expertise in Rehabilitation and Audiology, Hoensbroek, The Netherlands; 4 Department of Rehabilitation Medicine, Maastricht University, Maastricht, The Netherlands; 5 Department of Neuropsychology and Psychopharmacology, Faculty of Psychology and Neuroscience, Maastricht University, Maastricht, The Netherlands; 6 School for Mental Health and Neuroscience, Faculty of Health Medicine and Life Sciences, Maastricht University Medical Centre, Maastricht, The Netherlands

**Keywords:** Stroke, Geriatric rehabilitation, Elderly persons, Process evaluation

## Abstract

**Background:**

Almost half of the stroke patients admitted to geriatric rehabilitation has persisting problems after discharge. Currently, there is no evidence based geriatric rehabilitation programme available for older stroke patients, combining inpatient rehabilitation with adequate aftercare aimed at reducing the impact of persisting problems after discharge from a geriatric rehabilitation unit. Therefore, we developed an integrated multidisciplinary rehabilitation programme consisting of inpatient neurorehabilitation treatment using goal attainment scaling, home based self-management training, and group based stroke education for patients and informal caregivers. We performed a process evaluation to assess to what extent this programme was performed according to protocol. Furthermore, we assessed the participation of the patients in the programme, and the opinion of patients, informal caregivers and care professionals on the programme.

**Methods:**

In this multimethod study, process data were collected by means of interviews, questionnaires, and registration forms among 97 older stroke patients, 89 informal caregivers, and 103 care professionals involved in the programme.

**Results:**

A part of patients and informal caregivers did not receive all key elements of the programme. Almost all patients formulated rehabilitation goals, but among two thirds of the patients the goal attainment scaling method was used. Furthermore, the self-management training was considered rather complex and difficult to apply for frail elderly persons with stroke, and the percentage of therapy sessions performed in the patients’ home environment was lower than planned. In addition, about a quarter of the patients and informal caregivers attended the education sessions. However, a majority of patients, informal caregivers and care professionals indicated the beneficial aspects of the programme.

**Conclusion:**

This study revealed that although the programme in general is perceived to be beneficial by patients, and informal and formal caregivers, the feasibility of the programme needs further attention. Because of persisting cognitive deficits and specific care needs in our frail and multimorbid target population, some widely used methods such as goal attainment scaling, and self-management training seemed not feasible in their current form. To optimize feasibility of the programme, it is recommended to tailor these elements more optimally to the population of frail older patients.

## Background

The population of older stroke patients with persisting physical and psychosocial problems is rapidly increasing due to ageing of the population [1–4]. The prevalence of stroke among Dutch people of 65 years or older is estimated at 71 per 1000 males and 56 per 1000 females [5]. Almost half of the stroke patients admitted to geriatric rehabilitation has persisting problems after discharge such as paralysis, cognitive deficits, fatigue, behaviour problems and depression [6–12]. These problems might result in a decrease of the patient’s functional level, increased social isolation and can eventually result in admission to a long-term care facility. Furthermore, these problems may have a negative impact on the care burden and quality of life of their informal caregivers [13, 14]. This emphasizes the importance of continuity of care after home discharge of older stroke patients by providing adequate aftercare to prevent these problems.

In current stroke rehabilitation there is only limited attention for specialized aftercare to tackle and prevent further negative impact on patients and informal caregivers [15–17]. This indicates that it is important to improve stroke rehabilitation in providing more specialized aftercare which includes effective methods to increase the long term effects of stroke rehabilitation, and prevent or postpone admission to long term care facilities. However, there is no evidence based geriatric rehabilitation programme available for older stroke patients combining inpatient rehabilitation with adequate aftercare aimed at reducing the impact of persisting problems after discharge from a geriatric rehabilitation unit [18–31].

Therefore, we developed a multidisciplinary rehabilitation programme in which inpatient rehabilitation and after care are integrated. The new integrated programme is based on a combination of evidence available from stroke research about inpatient rehabilitation and aftercare, and expert knowledge from daily practice [17, 32]. The programme focusses on increasing the older stroke patient’s level of daily activity, functional independence, perceived quality of life, and social participation [32]. In addition, the programme aims to reduce the perceived burden of care and to increase the quality of life of the informal caregivers [32].

The effects of this newly developed rehabilitation programme have been evaluated by means of a multicentre randomized controlled trial with an intervention group receiving the new programme and a control group receiving usual care and will be reported elsewhere. The programme showed favourable effects on participation and autonomy of patients and on the care burden of informal caregivers.

Alongside this randomized controlled trial, we conducted a process evaluation to assess the feasibility of the program, based on the framework for process evaluation described by Saunders et al. [33] The current paper presents the results of this process evaluation of which the aims were: 1) to evaluate to what extent the integrated multidisciplinary rehabilitation programme was performed according to protocol (fidelity, dose delivered); 2) to evaluate the participation of the patients in the programme (reach and dose received exposure); and 3) to assess the opinion of patients, informal caregivers and care professionals on the programme (dose received satisfaction and context) [33]. More insight into these factors is relevant for both researchers and care professionals, because knowledge about the care processes could help to identify ways to optimize stroke rehabilitation for older persons and to set the agenda for future research [33, 34].

## Methods

### Design

This process evaluation study followed a multimethod design including qualitative and quantitative research methods (see Table 2). Process data were collected during a period of 12 months after patients were included in the rehabilitation programme. The study was conducted in the period of November 2010 and December 2015 with a total study period of 60 months. This process evaluation was conducted alongside a randomized controlled trial to evaluate the effectiveness of the newly developed multidisciplinary geriatric rehabilitation programme [32]. The randomized trial is registered by the following trial registration: International Standard Randomized Controlled Trial Register Number (ISRCTN62286281), and The Dutch Trial Register (NTR2412). This study is funded with a grant (grant number:313070301) from the Netherlands Organisation for Health Research and Development (ZonMw) as part of the National Care for the Elderly Programme.

### Integrated multidisciplinary rehabilitation programme

The programme, which was evaluated alongside the randomized controlled trial, consists of three care modules: 1) inpatient neurorehabilitation treatment; combined with 2 modules after discharge: 2) home based self-management training for patient and informal caregiver; and 3) stroke education for patient and informal caregiver. The intervention programme was delivered in eight geriatric rehabilitation units in the Netherlands. The programme was developed in close collaboration with members of the multidisciplinary stroke teams of the eight care organisations involved. Much effort was put in the implementation of the programme by training the care professionals in the study protocol, by periodical visits of the participating locations, and in being standby for tackling questions by the researchers of the study. The group of selected care professionals consisted of physical therapists, occupational therapists, speech therapists, psychologists, elderly care physicians, and stroke care coordinators. The main differences between the integrated multidisciplinary rehabilitation programme and usual care are presented in Table 1 and described in more detail below.

**Table 1:** Content differences between integrated multidisciplinary rehabilitation programme and usual care

	Integrated multidisciplinary programme	Usual care
***Care content***		
Multidisciplinary stroke team	+	+
Care based on Dutch stroke guidelines	+	+
Tailored approach with Goal Attainment Scaling	+	–
Self-management	+	–
Stroke education	+	–
Home therapy during nursing home admission	+	–
Multidisciplinary outpatient rehabilitation	+	–
Home visits of stroke care coordinator	+	–
***Care organisation***		
Stroke care coordinator	+	–
Multidisciplinary team meetings in nursing home	+	+
Multidisciplinary team meetings after discharge	+	–
Electronic patient record	+	–

#### Stroke care coordinator

In order to improve continuity of care, a stroke care coordinator was introduced as a member of the rehabilitation team. The stroke coordinator provides support to the patient and informal caregiver, facilitates the transition between rehabilitation and returning home and supports the collaboration between the care professionals involved.

#### Module 1: inpatient neurorehabilitation treatment

The first module starts when the patient is admitted to the geriatric rehabilitation unit and focuses on (re) learning the abilities needed to function as independently as possible in the home environment after discharge. At the start of this module, an individual treatment plan is developed with the patient. This treatment plan includes personal rehabilitation goals used during inpatient rehabilitation care and aftercare at the patient’s home. The method to formulate rehabilitation goals with the patient was a simplified version of the Goal Attainment Scaling (GAS) method [35, 36]. GAS has shown to be an appropriate method in rehabilitation treatment among elderly people [35, 36]. When the patient is admitted to the geriatric rehabilitation unit, the coordinator organizes an introductory meeting with the patient and primary informal caregiver. In this meeting, the coordinator provides general information about the rehabilitation programme. During their stay in the geriatric rehabilitation unit, the patient and an occupational therapist or physical therapist (depending on the individual care needs of the patients), visit the home of the patient to train with the patient in their own home environment. The therapist and patient use the home training sessions to train specific goals to increase and empower functional independence of the patient after discharge.

During one of the visits the therapists also check whether the patient’s home needs adjustments before discharge [37–39]. Additional financial means were arranged via the research project to facilitate the stroke team in organizing and performing these home therapy sessions (because travel expenses for therapist were not reimbursed in the regular reimbursement system). The care within this module is conducted by a multidisciplinary stroke team consisting of care professionals working at the geriatric rehabilitation unit including an a physical therapist, an occupational therapist, a speech therapist, a psychologist, an elderly care physician, and a stroke care coordinator.

To evaluate the treatment progress, multidisciplinary team meetings are organized every 4 weeks during the intervention period. To facilitate optimal communication and information transfer between care professionals, an electronic patient record is available for the primary and secondary care professionals involved in the programme. Furthermore, during the rehabilitation process the coordinator facilitates the transition of the patient from in-patient geriatric rehabilitation care to home-based (after) care by supporting the collaboration between the multidisciplinary stroke team of the geriatric rehabilitation unit, community health services and general practitioner. This module has a maximum duration of 2 months depending on the rehabilitation goals and care needs of the patient and informal caregiver.

#### Module 2: home based self-management training for patient and informal caregiver

The second module starts after discharge of the patient to the home environment and is focused on learning to cope with persisting cognitive and functional impairments as a result of stroke. To optimize the patient’s functional level and participation at least 50% of this module (i.e. therapy sessions with physical and/or occupational therapist) should be provided ambulatory in the home environment of the patient. The remaining part can be provided in an outpatient clinic. Furthermore, after discharge, the stroke coordinator conducts at least two home visits to the patient to support both patient and informal caregiver to improve their coping strategies. This training is based on strategies to enhance chronic disease self-management [40–42]. Furthermore, the stroke coordinator organizes multidisciplinary stroke team meetings with the primary care professionals involved. These meetings are aimed at evaluating the treatment process and to set rehabilitation goals for further treatment. The care in this module is provided by the same professionals of the multidisciplinary team of care professionals of the geriatric rehabilitation unit participating in module 1 and complemented with new care professionals from primary care. All care and support provided in this module is coordinated by the stroke coordinator under supervision of the general practitioner. This module has a maximum duration of 4 months, depending on the care needs of both patient and informal caregiver.

#### Module 3: stroke education for patient and informal caregiver

The third module is a stroke education module for patients and their informal caregivers. This module consists of four group sessions of 2 hrs (two mixed sessions with patients and informal caregivers and two sessions with patients and informal caregivers in separate groups) focusing on the psychological and emotional consequences of stroke, perceived problems in independent living and participation in society, and the role of the informal caregiver. The patients and informal caregivers are invited by the stroke care coordinator to participate in the course. The participation of the patient in the course should be planned within the intervention period of 6 months, and after patients were discharged home. The course is given by a (neuro) psychologist and two volunteers of the Dutch Stroke Patient Association and Informal Caregivers Association and a social worker.

#### Training care professionals

All care professionals of the participating stroke teams were trained in conducting the programme according to protocol. The training was conducted by members of the research team (TV, JvH and JV) during a 4 hrs session. The training consisted of interactive sessions about the key elements of the intervention, including the use of Goal Attainment Scaling (GAS), the training at the patients’ home, the use of self-management principles and the use of the electronic patient record. Care professionals who were not able to attend the training sessions received an individual session about the use of the protocol.

### Study population

The research population of the process evaluation consisted of three groups. The first group were 97 older stroke patients, who were allocated to the rehabilitation programme [32]. Patients were selected for participation in the present study when they met the following inclusion criteria: admission to one of the eight participating geriatric rehabilitation units located in the south of the Netherlands, due to a recent stroke, aged 65 or over, living independently in the community before the stroke, expected to be able to return home after discharge (as judged by the multidisciplinary stroke team), and giving informed consent to participate [32]. If the patient was unable to give informed consent, or the patient was medically unstable or had cognitive deficits and was not able to start rehabilitation on the basis of clinical judgment, the patient was excluded.

The second group consisted of 89 informal caregivers of the patients allocated to the rehabilitation programme. A person was considered to be the primary informal caregiver when the patient indicated him/her as the person from his social network who provides help with his or her activities of daily living, or instrumental activities of daily living on a long-term base. Informal caregivers could be included when they gave informed consent to participate in the study.

The third group consisted of 103 care professionals who participated in the eight stroke teams who conducted the rehabilitation programme. All participating care professionals were experienced in stroke rehabilitation and all care professionals who were involved in the treatment of patients in the intervention group were trained by a 3 h training in all key elements of the new rehabilitation programme. The stroke teams consisted of care professionals working at the participating geriatric rehabilitation units and community health care services, including elderly care physicians (*N* = 11), physical therapists (*N* = 24), occupational therapists (*N* = 18), speech therapists (*N* = 20), dieticians (*N* = 10), (neuro) psychologists (*N* = 7), and stroke care coordinators (*N* = 13).

### Measurement instruments

#### Patients and informal caregivers

The feasibility of the rehabilitation programme was assessed during a period of 12 months after the start of the programme for the individual patients (see Table 2). Process data from the patients were gathered by a trained research assistant by means of structured face-to-face interviews at 6 and 12 months, and after completion of module 3. Process data from the informal caregivers was gathered by a self-administered questionnaire at 6 and 12 months, and after completion of module 3. The data of module 3 was gathered on different time points because starting the module was dependent on the possibility to start module 3 with enough participants. On request of the informal caregiver, a research assistant could assist the informal caregiver in filling out the questionnaire.

**Table 2:** Outcome measures and measurement instruments of the process evaluation

Process outcomes	Patient	Informal caregiver	Care professionals		
	SI	SAQ	RF	SSQ	GI
**Performance of the programme according to protocol and participation in the programme**					
*Module 1: inpatient neurorehabilitation treatment for patients (2 months)*					
Development of rehabilitation goals			X	X	X
The use of the simplified goal attainment scaling method to set rehabilitation goals			X	X	X
Introduction meeting of stroke care coordinator			X	X	X
At least one home visit by 1) physical therapist and/or 2) occupational therapist to check for home adjustments			X	X	X
At least two therapy sessions in the patient’s home			X	X	X
*Module 2: home based self-management training for patient and informal caregiver (4 months)*					
Practicing self-management skills			X	X	X
Involving informal caregiver in self-management training			X	X	X
At least two home visits to the patient by the stroke care coordinator			X	X	X
At least 50% of the treatment sessions by 1) physical therapist and/or 2) occupational therapist at home			X		X
Number of patients and informal caregivers participating in the intervention group *(module 1 & 2)*			X		X
*Module 3: stroke education for patient and informal caregiver*					
Number of education sessions performed			X		X
Number of patients and informal caregivers attending the education sessions *(module 3)*			X		X
**Opinion of patients, informal caregivers and Care professionals on the programme**					
Patients’ and informal caregivers					
*Module 1: inpatient neurorehabilitation treatment for patients (2 months)*					
Perceived benefit of 1) setting rehabilitation goals, 2) therapy sessions in the patients’ home, 3) guidance of the stroke care coordinator	X	X			
*Module 2: home based self-management training for patient and informal caregiver (4 months)*					
Perceived benefit of 1) therapy sessions in the patients’ home, 2) home visits of the stroke care coordinator, 3) training self-management skills, 4) developing action plans to fulfil self-management training	X	X			
*Module 3: stroke education for patient and informal caregiver*					
Perceived benefit of the four education sessions	X	X			
*Care professionals*					
*Opinion multidisciplinary team*					
Benefit of 1) home visit to check whether home adjustments are needed *(module 1),* 2) the development of rehabilitation goals, 3) use of goal attainment scaling method *(module 1 & 2)*, 4) therapy sessions in the patients’ home *(module 2)*				X	X
*Opinion stroke care coordinator*					
Benefit of 1) development of rehabilitation goals *(module 2)*, 2) use of goal attainment scaling method *(module 2)*, 3) use of a workbook *(module 2)*, 4) practising self-management skills, 5) home visits after discharge *(module 2),* 5) personal guidance of the stroke care coordinator *(module 1 & 2)*, 6) four education sessions *(module 3).*				X	X

*SI* structured interview, *SAQ* self-administered questionnaire, *RF* research form, *SSQ* semi-structured questionnaire, *GI* group interview

#### Care professionals

Quantitative data concerning the implementation of the programme were gathered at the end of the randomized controlled trial by means of registration forms during the intervention period. The forms were included in the electronic patient records and were filled out by the care professionals who conducted the programme. In addition, a structured questionnaire (containing questions about the benefit of the key elements of the programme and opinion on the program) was sent after completion of the trial to all 103 care professionals who were involved in conducting the rehabilitation programme. The structured questionnaire had two versions; a version for the stroke care coordinators (*N* = 13) and a version for the stroke care team members (*N* = 90) (see Table 2 for further details on the contents of the questionnaire).

Furthermore, an additional group interview session with a small selection, of the 103 care professionals, with all involved care professionals of the stroke team represented, was scheduled within 3 months after data from the structured questionnaires were collected. Results from the questionnaires were used to select topics for the group interview. For the selection of care professionals for the interview the participating geriatric rehabilitation units were divided into two groups based on the number of participants in the programme during the study period of 48 months. One group consisted of the four geriatric rehabilitation units that included more than 30 patients in the rehabilitation programme. The second group consisted of the four geriatric rehabilitation units that included 30 patients or less in the rehabilitation programme. In both groups 10 care professionals working in community services and on a geriatric rehabilitation unit were selected by a purposive sampling method and invited to participate in the group interview. They were selected based on their experience with the programme. Both the invited groups consisted of stroke care coordinators (*N* = 3), an elderly care physician (*N* = 1), physical therapists (*N* = 2), an occupational therapist (*N* = 1), a speech therapist (*N* = 1), and neuropsychologists (*N* = 2). Both interviews had a planned duration of 1.5 h and were conducted by two researchers (TV and JvH). Both complete group interviews were audio recorded; a summary of the interviews was made by TV and JvH on the basis of the recording. The summaries were sent to the participating care professionals for confirmation (member check).

### Data analysis

Quantitative data from the structured interviews, self-administered questionnaires and registration forms were analysed by means of descriptive statistics using SPSS software package version 23 [32].

Qualitative data from the structured interviews, self-administered questionnaires, and group interview were classified into categories based on the given answers.

### Ethical considerations

The process evaluation was approved by the medical ethics committee of the University Hospital Maastricht and Maastricht University (MUMC+), the Netherlands. The alongside conducted randomized controlled trial is registered by the following registration numbers; International Standard Randomized Controlled Trial Register Number (ISRCTN62286281), and The Dutch Trial Register (NTR2412). Informed consent was obtained from all participating patients and informal caregivers.

## Results

### Response and background characteristics

Eighty four out of 97 patients (87%) participated in the interview after 6 months, and 70 patients (72%) participated in the interview after 12 months. Participating patients had a mean age of 78.8 years (SD = 6.3), an a mean activity level (FAI score) of 40.2 (SD = 8.8) a mean functional independence level (Katz-15 score) of: 6.0 (SD = 4.0), and mean cognitive score (MMSE-score: 21.9, SD = 5.2, threshold: ≤23.0). Regarding the informal caregivers, 68 informal caregivers out of 89 (76%) completed the questionnaire after 6 months, and 64 informal caregivers (71%) after 12 months. Participating informal caregivers had a mean age of 61.0 years (SD = 13.5), and a mean self-rated burden vas of 4.0 (SD = 2.4). Main overall reasons why patients and informal caregivers did not participate in the interviews were loss of interest (*N* = 6), lack of time (*N* = 3), an intercurrent illness (*N* = 4), or deceased (*N* = 7). Background characteristics of patients and informal caregivers are presented in Table 3.

**Table 3:** Background characteristics of included patients and informal caregivers

	Patients (***N*** = 97)	Informal caregivers (***N*** = 89)
*Characteristics*	*N (%)*	*N (%)*
Mean (SD) age	79 (7)^a^	61 (14)^a^
N (%) Female	69 (71)	53 (59)
Relationship with the patient		
- N (%) Spouse/partner	n.a.	28 (31)
- N (%) Family	n.a	59 (66)
- N (%) Friend	n.a.	2 (2)
- N (%) Other	n.a.	1 (1)
- N (%) No informal caregiver	n.a.	7 (7)

*n.a* not applicable

^a^ (SD)

A total of 59 care professionals (57%) responded to the questionnaire. The group care professionals, who responded, consisted of elderly care physicians (*N* = 2, 3%), physical therapists (*N* = 16, 27%), occupational therapists (*N* = 10, 17%), speech therapists (N = 12, 20%), neuropsychologists (*N* = 3, 5%), dieticians (*N* = 3, 5%), and stroke care coordinators (*N* = 13, 22%). The group interview was conducted with ten health professionals. All ten care professionals that were invited participated in the interview. The presented results of the interview were based on consensus of opinion within the group of care professionals who participated in the interview.

All care professionals who conducted the programme were experienced in stroke rehabilitation of elderly persons and were educated and trained in the relevant aspects of the intervention protocol.

### Performance according to protocol and participation in the programme

#### Module 1: inpatient neurorehabilitation treatment for patients

At baseline 97 patients were allocated to the intervention group and started with module 1 in the geriatric rehabilitation unit. After 6 months 11 patients had dropped out of the rehabilitation programme because of cognitive deficits (*N* = 3), loss of interest (*N* = 3), being deceased (*N* = 3) or other reasons (*N* = 2). The first module was conducted from 16 November 2010 until 4 December 2014.

In Table 4 the key components of the programme are presented. The multidisciplinary team developed with 94 (97%) of the 97 patient’s individual rehabilitation goals during inpatient and home based rehabilitation. During rehabilitation about two thirds (*N* = 60, 62%) of the patients developed rehabilitation goals with a care professional by using the goal attainment scaling (GAS) method.

**Table 4:** Performance according to protocol

Performance according to protocol^a^	
	N (%)
*Module 1: inpatient neurorehabilitation treatment for patients (2 months)*	
Number of patients started with the module	97 (100)
Number of informal caregivers started with the module	89 (100)
Development of rehabilitation goals with patient	94 (97)
The use of the goal attainment scaling method to set rehabilitation goals	60 (62)
Introduction meeting of stroke care coordinator with patient	96 (99)
At least one home visit by the physical therapist or occupational therapist to check for home adjustments	50 (52)
At least two therapy sessions by the physical therapist of occupational therapist in the patient’s home	11 (11)
*Module 2: home based self-management training for patient and informal caregiver (4 months)*	
Number of patients started with the module	86 (89)
Number of informal caregivers started with the module	74 (76)
Practicing self-management skills with the patient	53 (55)
Involving informal caregiver in self-management training of the patient	31 (32)
At least two home visits to the patient by the stroke care coordinator	60 (62)
At least 50% of the treatment sessions by physical therapist at home	38 (39)
At least 50% of the treatment sessions by occupational therapist at home	26 (27)
*Module 3: stroke education for patient and informal caregiver*	
*Patients*	
Number of patients participated	24 (25)
Mean number of sessions participated (out of a total of 4 sessions)	3.1
*Informal caregivers*	
Number of informal caregivers participated	23 (26)
Mean number of sessions participated (out of a total of 4 sessions)	3.1

^a^97 patients and 89 informal caregivers participated; ^a^ education sessions performed (%); ^b^ total amount of patients / total amount of informal caregivers participated in the intervention group

During the group interview there was consensus between the care professionals that setting rehabilitation goals by using the GAS method at the start of the rehabilitation was often difficult. Most participating care professionals mentioned that difficulties were often caused by limitations in communication skills of the patient and lack of insight in their disease. In those cases the therapist often set goals with the patient without using the GAS method. Almost all patients (*N* = 96, 99%) received an introduction meeting with the stroke care coordinator.

About half of the patients (*N* = 50, 52%) received at least one of the two home visits conducted by an occupational or physical therapist to practice in their own home environment and to check whether home adaptations should be made; 11 % (*N* = 11) of the patients received both therapy sessions at the patient’s home.

The group interview revealed that there was consensus between the therapists about the usefulness of home therapy, but it was often not performed because it was too time consuming due to travel distance.

Within the intervention period of 2 months 46 of the 97 patients (48%) were discharged home from the geriatric rehabilitation unit. However, almost half of the group (*N* = 51, 52%) was still not discharged because of complications that delayed the rehabilitation such as stroke recidivism, cardiac complication and delay in home adaptations or waiting for alternative accommodation. These patients continued module 1 awaiting to be discharged back home. The mean duration of stay in the rehabilitation unit was 83 days (range 7–456 days).

#### Module 2: home based self-management training for patient and informal caregiver

After discharge from the geriatric rehabilitation unit, all 86 patients who were still participating in the study continued the programme with module 2. Of the total group of patients (*N* = 86, 89%) who started module 2, 74 patients (86%) had an informal caregiver. The second module was conducted between 13 December 2010 and 14 December 2014.

Eighty-four patients of the total group of patients (*N* = 97) (87%) practiced self-management skills, of which 53 patients (55%) practiced self-management skills without their informal caregiver. These practice sessions were conducted at the patient’s home under guidance and supervision of the stroke care coordinator. During the interview with care professionals and the stroke care coordinators there was consensus about that training self-management skills was often too difficult for patients because it was complicated for them to develop and carry out action plans by themselves. In a lot of cases the therapists or stroke care coordinators had to set relevant and realistic goals with the patients because the patient was not capable of setting them by themselves.

In the intervention protocol it was planned that patients should receive a minimum of one home visit of the stroke coordinator to check how the patient and informal caregiver were doing at home. A total of 78 patients (80%) received at least one home visit and 60 patients (62%) received two or more home visits at the patient’s home. The number of home visits by the stroke care coordinator ranged between 1 and 5 visits, with a mean of 1.7 visits per patient.

For 39% of the patients (*N* = 38) at least half of the treatment sessions by the physical therapist was given at the patient’s home. In case of occupational therapy only 27% of the patients (*N* = 26) received therapy at home. The other treatment sessions were given in day treatment, practice or outpatient care setting. Most important reason why therapy was not conducted at home was that home therapy was considered very time consuming and costly.

All participating eight geriatric rehabilitation units organized a multidisciplinary meeting every 4 weeks for care professionals who were involved in the rehabilitation of the patients who were allocated to the intervention group. Five out of eight participating geriatric rehabilitation units used the for the intervention developed electronic patient record for communication between the care professionals. The reason for not using the electronic patient record was that these three organisations used another electronic patient record, which was not compatible with the study electronic programme. All patients completed this module within 4 months.

#### Module 3: stroke education for patient and informal caregiver

The patients who completed module 1 and 2 and thereafter still were in the study (*N* = 86) were invited for the four sessions of module 3. The information was handed out with further instruction and clarification by the stroke coordinator during a home visit with the individual patient and informal caregiver. Of the 86 patients who were invited to module 3, 68 (70%) agreed to participate and eventually 24 (25%) participated. The 24 patients who agreed to participate had a mean participation of 3.1 sessions. In total 64 of the 89 (72%) informal caregivers were invited, 23 (26%) informal caregivers participated with an average of 3.1 sessions. Main reason why patients (and related caregivers) not attended the sessions was because they were not interested in the sessions (*N* = 39), illness (*N* = 11), difficulties with transportation (*N* = 8), readmission to a geriatric rehabilitation unit (*N* = 5), too stressful (*N* = 4), on vacation (*N* = 3), work informal caregiver (*N* = 2), deceased (*N* = 2), and unknown (*N* = 11). Thirteen education sessions had to be cancelled because there were too few participants.

We planned four sessions per participating rehabilitation unit per every 6 months. Every cycle a group of 12 persons at the most (6 patients and 6 informal caregivers) was included. Taken the inclusion period and the amount of participating rehabilitation units (*N* = 8) into account we should have performed 15 education programmes of 4 sessions each, but eventually we only performed 6 education programmes of 4 sessions (40%) sessions. Main reason of the low number of sessions performed was the relatively low number of included participants per setting, which made it difficult to form groups and a lack of interest among the potential participants. Furthermore, the traveling distance to the sessions was in some cases a reason for not attending.

### Opinions on the programme

#### Patients

All patients who participated in the programme were asked to give their opinion on the key components of the programme they had received. The opinion of the patients on the different elements of the programme is presented in Table 5.

**Table 5:** Patients and informal caregivers’ perceived benefit of the programme^a^

Key components of the programme	N (%) patients who reported to have benefited from component		N (%) informal caregivers who reported to have benefited from component	
*Module 1: inpatient neurorehabilitation treatment for patients (2 months)*				
Setting rehabilitation goals with the Care professionals *(response patients N = 56)*	54	(96)	–	–
Therapy sessions in the patients’ home *(response patients N = 52)*	51	(98)	–	–
Guidance of the stroke care coordinator *(response patients N = 55 / informal caregivers N = 54)*	52	(95)	50	(93)
*Module 2: home based self-management training for patient and informal caregiver (4 months)*				
Home therapy sessions by a therapist *(response patients N = 46)*	43	(93)	–	–
Home visits of the stroke care coordinator *(response patients N = 52/ informal caregivers N = 50)*	47	(90)	43	(86)
Setting goals for training self-management skills *(response patients N = 35 / informal caregivers N = 46)*	34	(97)	40	(87)
Developing action plans to fulfil self-management training *(response patient N = 32 / informal caregivers N = 42)*	30	(94)	38	(90)
*Module 3: stroke education for patient and informal caregiver*				
Four education sessions (module 3) *(response patients N = 24 / informal caregivers N = 28)*	22	(92)	27	(96)

^a^Measured in patients and informal caregivers who actually did received the key elements of the programme

Of the 56 patients who followed module 1 and formulated goals with the care professionals, 54 patients (96%) indicated that they benefited from it. Almost all patients (98%) of the patients (*N* = 51) who actually did receive home therapy reported to have benefited from these therapy sessions. From the patients who received module 2 and trained self-management skills by setting goals also almost all patients (*N* = 34, 97%) indicated that they had benefited from this key element of the programme.

Patients who participated in the rehabilitation programme were asked how the programme could be improved. They indicated that the programme could be improved by providing more information about the program itself to the participants, increasing the support patients receive from the stroke coordinator and providing more information to the patients about the roles of the different care professionals who perform the programme.

#### Informal caregivers

Of the informal caregivers of which the patient actually followed module 1, 93% (*N* = 50) perceived benefit of the support of the stroke care coordinator. Eighty-seven percent of the informal caregivers (*N* = 40) of which the patient followed module 2 perceived benefit of goal setting for training self-management skills and 90% of the informal caregivers (*N* = 38) benefited from developing action plans to fulfil self-management training.

The informal caregivers were asked how the programme could be improved. They made the following suggestions: more focus on the necessary home adaptions to facilitate a fast transfer back home, more personal support from the care coordinator during admission, better and faster continuation of the programme after discharge home.

#### Care professionals

The opinion of the 34 care professionals and 13 stroke coordinators who responded and filled in the questionnaire is presented in Table 6. Thirty-three (97%) of the 34 care professionals who conducted all modules of the programme indicated that patients did benefit from the development of rehabilitation goals and 30 care professionals (91%) considered the use of the goals attainment scaling method to be beneficial for patients and informal caregivers. However, the self-management method which was used to stimulate patients in their problem-solving skills was perceived rather complex and difficult to apply. They considered it important to make this method more accessible for this frail population to improve its feasibility.

**Table 6:** Care professionals’ opinion about the benefit of the programme for patients and informal caregivers^a^

Key components of the programme	N (%) Care professionals who reported that component is beneficial for patient and/or informal caregivers
*Opinion multidisciplinary team (without stroke coordinator) (response N = 48)*	
Development of rehabilitation goals with the patient *(module 1 & 2) (response N = 36)*	33 (97)
Use of goal attainment scaling method to develop rehabilitation goals *(module 1 & 2) (response N = 33)*	30 (91)
Home visit to check whether home adjustments are needed *(module 1) (response N = 19)*	14 (74)
Therapy sessions in the patients’ home *(module 2) (response N = 23)*	20 (95)
*Opinion stroke care coordinator (response N = 13)*	
Development of rehabilitation goals with the patient (module 2)	12 (92)
Use of goal attainment scaling method to develop rehabilitation goals (module 2)	11 (85)
Use of a workbook to develop rehabilitation goals and action plans (module 2)	9 (69)
Practicing self-management skills with the patient and informal caregiver *(module 2)*	9 (69)
Home visits after discharge *(module 2)*	12 (92)
Personal guidance of the stroke care coordinator *(module 1 & 2)*	12 (92)
Four education sessions *(module 3)*	9 (69)

^a^Measured among members of the multidisciplinary team and stroke coordinators who conducted the key elements of the programme

The stroke care coordinators were unanimously in their opinion about the benefits of developing rehabilitation goals, home visits after discharge and their personal guidance at home.

The results of the group interviews indicated that the education sessions should be changed on a few points. The group suggested to start with the sessions when patients are still at the geriatric rehabilitation unit, and combine the sessions with a training activity such as for example exercising with a physical therapist. Furthermore, in their opinion the group should not include more than maximum 10 patients. A larger group could lead to less interaction between the group members and information loss.

Both care professionals and stroke care coordinators mentioned that multidisciplinary team meetings and using an electronic patient record are important tools to optimize communication during rehabilitation. Finally, recommendations were made to continue the programme without the element of home visits to check for home adaptations and train with the patient at home, because of the time consumption and financial limitations. The role of the stroke care coordinator was indicated as very important and should be continued according to the care professionals. Facilitating further aftercare and guiding stroke patients and informal caregivers after discharge could be very important to prevent decline in functioning of the patient and admission in a long term care facility.

## Discussion

This study evaluated the feasibility of an integrated multidisciplinary stroke rehabilitation program, for older persons (65+) who suffered a stroke. The study revealed that the program was conducted only partly according to protocol. A substantial part of patients and informal caregivers did not receive all key elements of the three care modules of the program. Almost all patients formulated rehabilitation goals and received an introduction meeting of the stroke care coordinator, but not for all patients the goal attainment scaling method was used to set these rehabilitation goals. Although goal attainment scaling is the recommended method for setting rehabilitation goals in stroke, its feasibility for this frail older population of stroke patients is limited according to the care professionals. Our results showed that most difficulties were caused by limitations in communication skills of the patient and lack of insight in their disease. Furthermore, the self-management method used to stimulate patients in their problem-solving skills was considered rather complex and difficult to apply for frail elderly persons with stroke, by the care professionals. This could be due to different reasons. First, the capabilities of these frail older persons to process new information are often more limited than in younger stroke patients. Second, it could be that our training of the participating care professionals in learning to teach self-management principles during a training of 4 hrs might have been too short. To increase the application of self-management by care professionals in stroke care it might be necessary to give more intensive training during a longer period of time. Furthermore, self-management strategies were introduced relatively early after stroke. Moulaert et al., also experienced in a study among patients who survived cardiac arrest, that self-management strategies were difficult to implement as an early intervention in the first weeks of rehabilitation [43]. Maybe using this method in the chronic phase of stroke could lead to more effective use.

Furthermore, it was observed that although additional financial means were made available for home visits, the amount of home visits to check for necessary adaptations and the conduct of therapy sessions in the patients’ own home environment was lower than planned. Thus, enhancing financial possibilities seems not to be enough in lowering the barrier for therapists to conduct home visits. Main barriers brought up during our group interview with the stroke professionals were time and travel problems. Although, home therapy is valued for its effect to enhance the functional activity and participation level of stroke patients after discharge [44], results of our study indicate that there is still a time and/or travel problem for care professionals which makes it is still rather difficult, in current practice, to organize home therapy. To tackle time and travel difficulties, telemonitoring could be a feasible and effective alternative to improve recovery, and maintaining the benefits reached during inpatient rehabilitation [45–47].

Finally, we also observed that the attendance of the education module by the patients and informal caregivers was rather low. It seemed to be difficult to motivate older stroke patients and their informal caregivers to visit education sessions about stroke after discharge, and it remains unclear how to improve the feasibility of this module. The majority of the people who declined to participate in this module indicated they were not interested in participation. However, there might be some underlying reasons for this lack of interest which we concluded from our study, such as perceived burden and practical and financial concerns related to travelling to the location. Therefore, providing this module in a more accessible way, such as in the form of written information and/or video education such as telemonitoring, could be considered [45–47].

This study has several limitations. First, there is always a risk that the results of the questionnaires are biased by socially desirable answers from the participating patients, informal caregivers and care professionals. To reduce this risk of bias a research assistant conducted the questionnaires in case of the patients and informal caregivers and the care professionals received the questionnaires by mail to ensure they filled out the questionnaire without the presence of the researcher. The data of the questionnaires were processed anonymously, but this does not completely eliminate the chance of bias in our data.

Second, the response rate of the care professionals was limited so it is unclear whether the answers are representative for the total group. This might be due to high workload.

Third, we conducted a group interview with a selection of care professionals but not with a selection of patients and informal caregivers. A group interview with patients and informal caregivers might have provided important additional information on how to improve the intervention.

An important strength of our study is the broad approach of evaluation, which gathered data from care professionals as well as patients and informal caregivers. This study is one of the first in evaluating stroke rehabilitation in older stroke patients.

Despite our intensive collaboration with the stroke care field in developing the program, the results of our process evaluation show that the program was only partly feasible. Implementation research shows that implementing complex interventions like ours is very challenging in an older population [48]. It seems that complex interventions such as this in clinical practice for older stroke patients require a more intensive and stepwise implementation strategy such as described by Luker and Dowding [48, 49] This method could be important to increase necessary knowledge of the key elements in the treatment protocol and enhance the collaboration between members of the rehabilitation teams. Furthermore, we recommend to assess the feasibility and effectiveness of this type of complex interventions by using action research, which gives the researcher the possibility to expand pilot testing including a cyclical and flexible process to optimize the intervention during the research period which is not possible when a process evaluation is performed alongside a randomized controlled trial as was the case in our study [49, 50].

## Conclusion

This study revealed that the feasibility of the new rehabilitation programme needs further attention. Because of the persisting cognitive deficits and specific care needs in our target population some methods such as goal attainment scaling, self-management training and stroke education seemed not feasible in its current form. To optimize feasibility, these elements could be simplified to make them more suitable for the rehabilitation of older patients. In addition, training of care professionals could be improved. We expect that increasing the feasibility of the programme could also further increase its effectiveness. In addition, the action research method could be a useful tool to tailor the programme optimally to the care setting, care professionals and patients involved.

## Data Availability

The datasets used and/or analysed during the current study are available from the corresponding author on reasonable request.
